# U-73122, a phospholipase C inhibitor, impairs lymphocytic choriomeningitis virus virion infectivity

**DOI:** 10.1099/jgv.0.002060

**Published:** 2024-12-17

**Authors:** Keita Mizuma, Mei Hashizume, Shuzo Urata, Keiko Shindo, Ayako Takashima, Satoshi Mizuta, Masaharu Iwasaki

**Affiliations:** 1 Laboratory of Emerging Viral Diseases, International Research Center for Infectious Diseases, Research Institute for Microbial Diseases, Osaka University, Suita, Osaka, Japan; 2 National Research Center for the Control and Prevention of Infectious Diseases, Nagasaki University, Nagasaki, Japan; 3 Center for Bioinformatics and Molecular Medicine, Graduate School of Biomedical Sciences, Nagasaki University, Nagasaki, Japan; 4 Center for Infectious Disease Education and Research, Osaka University, Suita, Osaka, Japan; 5 Center for Advanced Modalities and Drug Delivery System, Osaka University, Suita, Osaka, Japan; 6 RNA Frontier Science Division, Institute for Open and Transdisciplinary Research Initiatives, Osaka University, Suita, Osaka, Japan

**Keywords:** LCMV, antiviral, virion, *Lassa virus*, *Junín virus*

## Abstract

Lassa virus (LASV) is an Old World (OW) mammarenavirus that causes Lassa fever, a life-threatening acute febrile disease endemic in West Africa. Lymphocytic choriomeningitis virus (LCMV) is a worldwide-distributed, prototypic OW mammarenavirus of clinical significance that has been largely neglected as a human pathogen. No licensed OW mammarenavirus vaccines are available, and the current therapeutic option is limited to the off-label use of ribavirin, which offers only partial efficacy. This situation underscores the urgent need to develop novel antivirals against human pathogenic mammarenaviruses. Previously, we showed that afatinib, a pan-ErbB tyrosine kinase inhibitor, inhibited multiple steps of the life cycles of OW LASV and LCMV, as well as the New World Junín virus vaccine strain Candid#1. In the present study, we investigated the inhibitory effect of U-73122, a phospholipase C inhibitor that acts downstream of ErbB signalling, on LCMV multiplication. U-73122 inhibited WT recombinant (r) LCMV multiplication in cultured cells. Preincubation of cell-free LCMV virions with U-73122 resulted in impaired virion infectivity. U-73122 also inhibited the infection of rLCMVs expressing heterologous viral glycoproteins, including the vesicular stomatitis Indiana virus (VSIV) glycoprotein, whereas WT VSIV infection was not affected by U-73122 treatment. Our results show the novel bioactivity of U-73122 as an LCMV inhibitor and indicate the presence of a virion-associated molecule that is necessary for virion infectivity and can be exploited as a potential antiviral drug target against human pathogenic mammarenavirus infections.

## Introduction

Several mammalian arenaviruses (mammarenaviruses) cause haemorrhagic fever diseases in humans and pose significant public health concerns in their endemic regions [1,5]. Lassa virus (LASV) is a highly prevalent Old World (OW) mammarenavirus and the causative agent of the life-threatening acute febrile disease Lassa fever [5]. LASV is estimated to infect several hundred thousand individuals in West Africa every year, resulting in a high number of Lassa fever cases associated with high levels of morbidity and mortality [6,9]. The regions of LASV endemicity are expanding [9,11], and increased travel has resulted in Lassa fever cases being imported into nonendemic metropolitan areas [12,14]. Furthermore, the worldwide-distributed, prototypic mammarenavirus, lymphocytic choriomeningitis virus (LCMV), is a human pathogen of clinical significance, especially in immunocompromised individuals or cases of congenital infection [15,19]. Despite their significant impact on human health, there are no licensed vaccines against OW mammarenaviruses, and current anti-mammarenavirus treatment is limited to the off-label use of ribavirin, which is only partially effective and associated with significant side effects [20,22]. Therefore, there is an urgent need for the development of novel antivirals against human pathogenic mammarenaviruses.

Mammarenaviruses are enveloped viruses with a bi-segmented, ssRNA genome [23]. Genome segments S and L use an ambisense coding strategy to direct the expression of two viral genes arranged in opposite orientations, separated by a noncoding intergenic region. The S segment encodes the viral nucleoprotein (NP) and glycoprotein precursor (GPC). The GPC is co-translationally processed by a signal peptidase to generate a stable signal peptide and post-translationally processed by cellular proprotein convertase subtilisin kexin isozyme-1/site 1 protease (SKI-1/S1P) to generate the mature virion surface glycoproteins GP1 and GP2. GP1 and GP2 together with the stable signal peptide form the glycoprotein complex that is responsible for receptor recognition and cell entry. The L segment encodes viral RNA-dependent RNA polymerase (L) and small RING finger protein (Z). NP and L protein together with the genome RNA form the viral ribonucleoprotein (vRNP) complex that is responsible for directing the replication and transcription of the viral genome [24]. The LASV virion attaches to a cell surface receptor, primarily *α*-dystroglycan (*α*DG), and enters the cell via an atypical pathway of macropinocytosis [25,26]. When the virus reaches the acidic environment of the endosome, GP1 undergoes a conformational change that allows it to bind to the intracellular receptor LAMP1 [27]. This is followed by membrane fusion between viral and cellular membranes induced by a conformational change of GP2, releasing vRNP into the cell cytoplasm where viral gene expression and genome replication take place. CD164, an essential entry factor for LCMV [28,29], may be an analogue for LAMP1 that facilitates LASV cell entry because CD164 was required to trigger LCMV glycoprotein-mediated membrane fusion at a low pH, which mimics the acidic milieu of the endosome where LCMV membrane fusion takes place [28]. Eventually, infectious progeny virions are assembled and released from the plasma membrane by Z-mediated budding.

A major obstacle in the development of anti-LASV drugs is the requirement of biosafety level 4 containment for handling infectious LASV. To address this issue, we previously used a minigenome assay-based, infectious-free cell platform that recreated a vRNP of LASV, where the levels of vRNP-directed reporter gene expression served as a surrogate for vRNP activity [30], which we used to screen a US Food and Drug Administration (FDA)-approved drug library [31]. We identified afatinib, an inhibitor of pan-ErbB receptor tyrosine kinase, as a potent inhibitor of LASV vRNP activity. Afatinib also inhibited the multiplication of the OW mammarenavirus, LCMV, and New World (NW) Junín virus (JUNV) vaccine strain Candid#1. JUNV is a causative agent of Argentinian haemorrhagic fever, a disease endemic in the Pampas region of Argentina with a high mortality rate [32,33]. Cell-based assays showed that afatinib inhibited multiple steps of the life cycles of LASV, LCMV and JUNV, including cell entry, transcription and replication of the viral genome and budding. Using several inhibitors for pathways downstream of ErbB signalling, we found that the PI3K/AKT and JAK/STAT pathways and ERK/MAPK and JAK/STAT pathways contributed to LASV and LCMV vRNP activity, respectively. Interestingly, a small molecule compound, U-73122, which inhibits phospholipase C (PLC)-*γ* [34,36] or dasatinib, an Src inhibitor, did not have a deleterious impact on LASV or LCMV vRNP activity, implying that downstream pathways of ErbB signalling, the inhibitors of which did not inhibit vRNP activity, may promote biological processes other than vRNP activity during mammarenavirus multiplication. In the present study, we investigated the potential inhibitory effect of U-73122 on LCMV multiplication.

## Methods

### Cells and viruses

HEK293T [American Type Culture Collection (ATCC); CRL-3216], Vero E6 (ATCC; CRL-1586) and A549 (ATCC; CCL-185) cells were cultured in Dulbecco’s modified Eagle medium (DMEM) (FUJIFILM Wako Pure Chemical Corporation) containing 10% heat-inactivated FBS, 100 U ml^−1^ penicillin and 100 µg ml^−1^ streptomycin (10% FBS/DMEM) at 37 °C and 5% CO_2_. BHK-21 (ATCC; CCL-10) cells were cultured in 10% FBS/DMEM supplemented with 5% tryptose phosphate broth at 37 °C and 5% CO_2_.

WT recombinant (r) clone 13 (rCl-13), WT rArm, rCl-13 expressing ZsGreen (rCl-13-ZsG), rCl-13 expressing LASV GPC instead of rCl-13 GPC and rCandid#1 were generated by reverse genetics as described previously using BHK-21 cells [37,40]. To generate rCl-13-ZsG/Can1GPC and rCl-13-ZsG/VSIV-G, plasmids mPol1Sag(Can1GPC/ZsG-P2A-NP) and mPol1Sag(VSIV-G/ZsG-P2A-NP) were generated by replacing the GPC ORF in mPol1Sag(GPC/ZsG-P2A-NP) that directs the murine polymerase I-mediated intracellular synthesis of S RNA anti-genome species and were used to generate rCl-13-ZsG by the Candid#1 GPC or the vesicular stomatitis Indiana virus glycoprotein (VSIV-G) ORF, respectively. Rescue of rCl-13-ZsG/Can1-GPC and rCl-13-ZsG/VSIV-G was as described for rCl-13-ZsG using plasmids mPol1Sag(Can1GPC/ZsG-P2A-NP) and mPol1Sag(VSIV-G/ZsG-P2A-NP) instead of plasmid mPol1Sag(GPC/ZsG-P2A-NP), respectively. Vesicular stomatitis Indiana virus (VSIV) was obtained from Professor Kida (Hokkaido University, Sapporo, Hokkaido, Japan) [41].

### Virus titration

LCMV titres were determined using an immunofocus-forming assay described previously [42] with minor modifications. Vero E6 cells seeded in 96-well plates at 2.0×10^4^ cells/well and cultured overnight were inoculated with tenfold serial dilutions of LCMV. After 20-h incubation at 37 °C and 5% CO_2_, the cells were fixed with 4% paraformaldehyde (PFA) in PBS (4% PFA/PBS; Nacalai Tesque). To visualize cells infected with rLCMVs that did not possess the ZsG gene, the cells were permeabilized with 1% normal goat serum (Nacalai Tesque) in dilution buffer (0.3% Triton X-100 in PBS-containing 3% BSA) and stained with a rat monoclonal antibody to NP (VL-4; Bio X Cell) conjugated with Alexa Fluor 488 (VL-4-AF488; Alexa Fluor 488 Protein Labelling Kit; Thermo Fisher Scientific). Fluorescent images were captured with a CQ1 confocal quantitative image cytometer (Yokogawa Electric Corporation), and the NP-positive or ZsG-positive LCMV-focus numbers were determined using the high-content analysis software CellPathfinder (Yokogawa Electric Corporation). Virus titres were calculated by multiplying the NP-positive or ZsG-positive LCMV-focus number by the corresponding dilution factor. HEK293T cells were used instead of Vero E6 cells to determine rCl-13-ZsG/Can1-GPC and rCandid#1 titres. Titres of VSIV were determined by a plaque assay using Vero E6 cells.

### Compounds

U-73122 (CAS#112648-68-7) was purchased from MedChemExpress or Selleck Chemicals. U-73343 (CAS#142878-12-4) was purchased from MedChemExpress.

### Determination of 50% effective concentration in the cell-based rLCMVs expressing ZsG infection assay

HEK293T cells (4.5×10^4^ or 7.5×10^4^ cells/well) or A549 cells (2×10^4^ cells/well) seeded in a 96-well plate and cultured overnight were treated with threefold serial compound dilutions at 37 °C and 5% CO_2_ for 1.5 h or remained untreated. An rLCMV expressing ZsG was added (m.o.i.=0.1 or 0.01) to freshly prepared threefold serial compound dilutions, and the virus-compound mixtures were added to the cells. After 1 h of virus adsorption, the virus inoculum was removed, the cells were washed with PBS and a fresh medium with or without the compound dilutions was added. Alternatively, rCl-13-ZsG was added (m.o.i.=0.01) to the cells cultured in the medium containing compound. At 48 h post-inoculation (hpi), the cells were fixed with 4% PFA/PBS. The ZsG signal intensity was measured using a multi-mode microplate reader (SpectraMax iD5; Molecular Devices). The mean ZsG signal intensity of vehicle (DMSO)-treated and virus-infected cells was set to 100%. The 50% effective concentration (EC_50_) values were determined using GraphPad Prism (GraphPad Software).

### Determination of 50% cytotoxic concentration in the cell-based rLCMVs expressing ZsG infection assay

HEK293T cells (4.5×10^4^ or 7.5×10^4^ cells/well) or A549 cells (2×10^4^ cells/well) seeded in a 96-well plate and cultured overnight were treated with threefold serial compound dilutions at 37 °C and 5% CO_2_. In several experiments, compounds were removed after 1 h of incubation and fresh media were added. After 48 h, CellTiter 96 AQueous One Solution Reagent (Promega) was added. Thereafter, the assay was performed according to the manufacturer’s recommendations. Absorbance (490 nm) was obtained using a multi-mode microplate reader (SpectraMax iD5). The mean value obtained from DMSO-treated cells was set to 100%. The 50% cytotoxic concentration (CC_50_) values were determined using GraphPad Prism (GraphPad Software).

### Time-of-addition experiment

HEK293T cells (2.5×10^5^ cells/well) seeded in a 24-well plate and cultured overnight were treated with U-73122 (5 µM), NH_4_Cl (20 mM) or vehicle (DMSO) for various periods: −1.5 to 0 (Pre), 0 to 1 (During) or 1 to 24 (Post) hpi (m.o.i.=0.1) with rCl-13-ZsG or WT rCl-13. Freshly prepared medium containing U-73122, NH_4_Cl or vehicle was added to some cells at −1.5, 0 and 1 h post-virus inoculation, and the cells were cultured until 24 hpi (throughout, Thrut). Virus inoculum was prepared in the medium containing the compounds or vehicle (DMSO). At 24 hpi, rCl-13-ZsG-inoculated cells were fixed, and ZsG expression was observed using a fluorescence microscope (ECLIPSE Ti2-U; Nikon). At 24 hpi, WT rCl-13-inoculated cells were collected using Accutase cell detachment solution (Nacalai Tesque), and the proportions of virally infected cells were analysed by flow cytometry.

### Flow cytometry

Cells were fixed with 4% PFA/PBS and permeabilized and blocked with dilution buffer. The cells were then stained with VL-4-AF488. LCMV NP expression was examined using a cell sorter (SH800H; SONY), and the data were analysed using Cell Sorter software (SONY).

### Statistical analysis

GraphPad Prism 9 (GraphPad Software) was used for all the statistical analyses. Statistical significance was analysed by one-way ANOVA, and statistically significant differences were determined by Tukey’s multiple comparison test.

## Results

### U-73122 inhibits LCMV multiplication

**Fig. 1.**
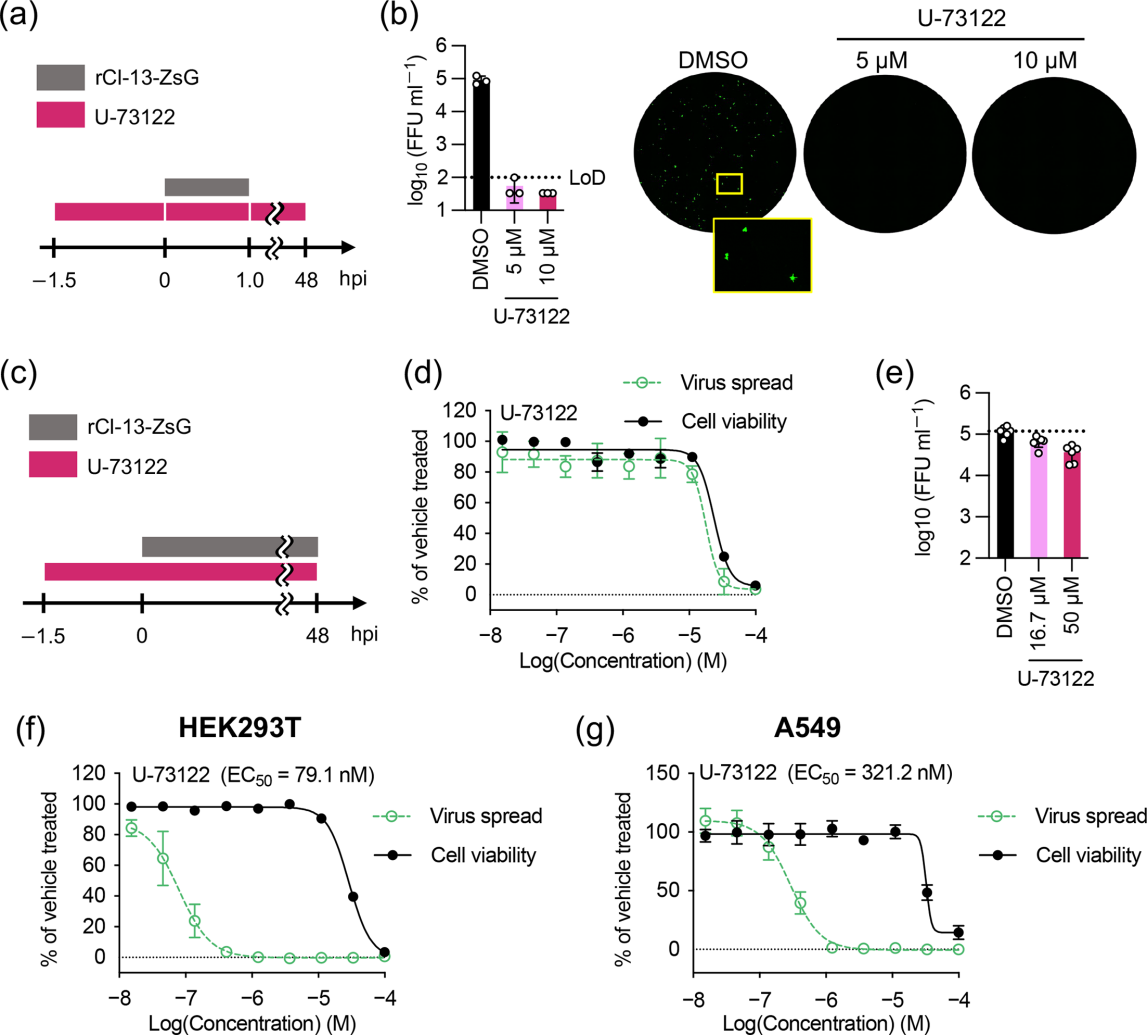
Effect of U-73122 on rCl-13 multiplication. (**a**) Schematic diagram of the duration of U-73122 treatment and rCl-13-ZsG inoculation in the experiments of (b), (f) and (g). (**b**) rCl-13-ZsG titres in TCSs of cells treated with U-73122. HEK293T cells seeded in a 24-well plate (2.5×10^5^ cells/well) and cultured overnight were treated with U-73122 or DMSO and inoculated (m.o.i.=0.01) with rCl-13-ZsG as indicated in (a). Virus titres in TCSs collected at 48 hpi were determined by immunofocus-forming assay (IFFA). FFU, focus-forming unit; LoD, limit of detection. Data are the mean±sd of three independent experiments. Representative images of the infectious foci are shown on the right. The zoomed image and corresponding area in the well image are marked by yellow boxes. (**c**) Schematic diagram of the duration of U-73122 treatment and rCl-13-ZsG inoculation in the experiments of (d) and (e). (**d, e**) Determination of the EC_50_ and CC_50_ values. HEK293T cells seeded in a 96-well plate (7.5×10^4^ cells/well) and cultured overnight were treated with threefold serial dilutions of U-73122 or with DMSO and inoculated (m.o.i.=0.01) with rCl-13-ZsG as indicated in (c). At 48 hpi, cells were fixed to examine ZsGreen expression (virus spread). Cell viability of U-73122-treated and uninfected HEK293T cells was determined using CellTiter 96 AQueous One Solution Reagent. (**e**) Viral titres in TCSs of some cells collected before fixation were determined by IFFA. (**f**) EC_50_ and CC_50_ values were determined as in (d), but treating and inoculating cells with U-73122 and rCl-13-ZsG for the durations indicated in (a). (**g**) EC_50_ and CC_50_ values were determined as in (f), except that A549 cells were used instead of HEK293T cells. Data are the mean±sd of six (d–g) replicates.

In a previous study, we showed that U-73122 did not inhibit the minigenome activity of LASV or LCMV [31]. To determine whether U-73122 inhibited a distinct step of the mammarenavirus life cycle other than the transcription or replication of the viral genome, we investigated the effect of U-73122 on mammarenavirus multiplication using an rCl-13 variant of the Armstrong (Arm) strain of LCMV expressing ZsG (rCl-13-ZsG). We treated HEK293T cells with U-73122 (5 or 10 µM) or vehicle (DMSO). After 1.5 h, cell culture medium [tissue culture supernatant (TCS)] was removed, the cells were washed with PBS, and virus inoculum containing U-73122 or vehicle was added to the cells (m.o.i.=0.01). After 1 h of virus adsorption, the cells were washed with PBS, and a fresh medium containing U-73122 or vehicle was added. At 48 hpi, the virus titres of TCSs were determined (Fig. 1a). U-73122 aborted the production of infectious rCl-13-ZsG progeny (Fig. 1b). To assess the correlation between the efficacy and cytotoxicity of U-73122, we determined the selectivity index (SI=CC_50_/EC_50_) of U-73122. To determine EC_50_, HEK293T cells were treated with threefold serial dilutions of U-73122 or vehicle (DMSO). After incubation for 1.5 h, rCl-13-ZsG inoculum was added to the cells. At 48 hpi, TCSs were collected, and the cells were fixed. Virus propagation was assessed by the ZsG signal intensity (Fig. 1c). Whereas U-73122 strongly inhibited rCl-13-ZsG progeny production (Fig. 1b), U-73122 did not inhibit ZsG signal intensity levels at concentrations that did not affect cell viability (Fig. 1d). In addition, viral titres of TCSs were not reduced by the U-73122 treatment at concentrations as high as 50 µM (Fig. 1e). A possible explanation for this discrepancy is that U-73122 may have lost its inhibitory effect on LCMV multiplication by the time of virus addition in the latter experiment. To test this hypothesis, EC_50_ was determined with a protocol that was similar to the one used for the former experiment (Fig. 1a). With this protocol, we confirmed the dose-dependent inhibitory effects of U-73122 within the concentration range that had negligible impact on cell viability; U-73122 had an SI of 359 (CC_50_=28.4 µM, EC_50_=79.1 nM) (Fig. 1f). To examine whether the anti-LCMV activity of U-73122 was specific to the infection of HEK293T cells, we used A549 cells and determined the EC_50_. U-73122 also inhibited rCl-13-ZsG infection of A549 cells in a dose-dependent manner with an SI of 103 (CC_50_=33.1 µM, EC_50_=321.2 nM) (Fig. 1g).

### U-73122 inhibits an early step of the LCMV life cycle

**Fig. 2.**
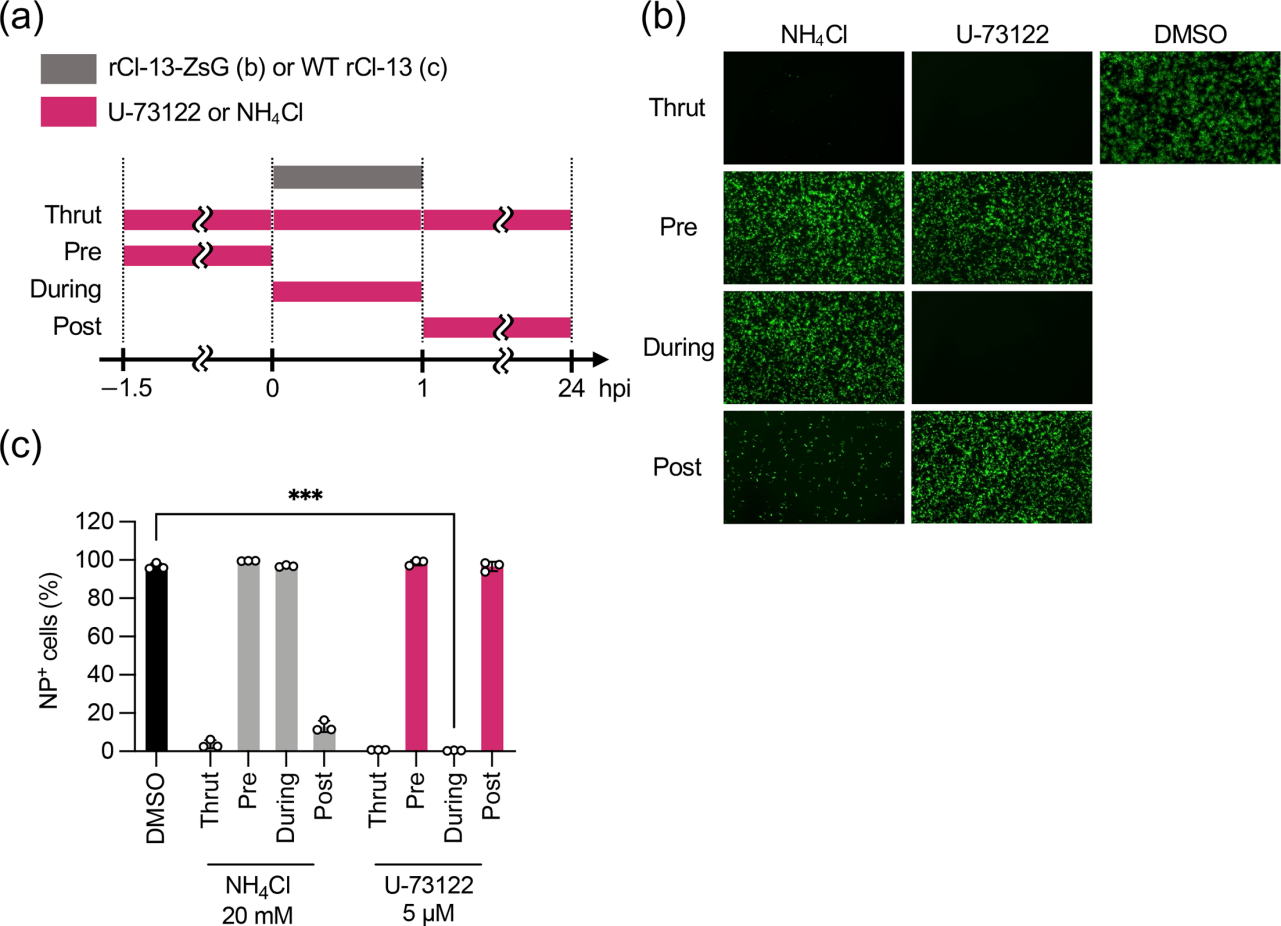
Effect of U-73122 on an early step of LCMV multiplication. (**a**) Schematic diagram of the duration of U-73122 treatment and rCl-13-ZsG or WT rCl-13 inoculation. (**b, c**) HEK293T cells seeded in a 24-well plate (2.5×10^5^ cells/well) and cultured overnight were treated with 20 mM ammonium chloride (NH_4_Cl), 5 µM U-73122 or DMSO during the indicated time points and inoculated (m.o.i.=0.1) with rCl-13-ZsG (b) or WT rCl-13 (c) as indicated in (a). At 24 hpi, ZsG expression was visualized by fluorescent microscope (**b**) or LCMV-NP-positive (NP^+^) cells were detected by flow cytometry (**c**). Data are the mean±sd of three independent experiments. ***P<0.001.

U-73122 strongly inhibited LCMV multiplication with a relatively short duration of efficacy, implying that U-73122 blocked an early step of the viral life cycle. To examine this possibility, we performed a time-of-addition experiment. HEK293T cells were treated with U-73122 (5 µM), ammonium chloride (NH_4_Cl; 20 mM) or vehicle (DMSO) for −1.5 to 0 h (Pre), 0 to 1 h (During) or 1 to 24 h (Post) post-rCl-13-ZsG inoculation (m.o.i.=0.1) (Fig. 2a). U-73122, NH_4_Cl or vehicle was added to some cells at −1.5, 0, and 1 h post-virus inoculation, and the cells were cultured until 24 hpi (throughout, Thrut). NH_4_Cl was used as a positive control to block LCMV cell entry because it increases endosomal pH and prevents the low-pH-dependent fusion event between viral and cellular membranes required for the completion of cell entry [43]. As expected, Thrut-NH_4_Cl treatment almost completely inhibited rCl-13-ZsG infection, and the slight increase of virally infected (ZsG-positive) cells observed in Post-NH_4_Cl-treated cells suggested that NH_4_Cl blocked secondary infection of progeny virions produced from cells infected with rCl-13-ZsG before the initiation of NH_4_Cl treatment (Fig. 2b). No inhibition of ZsG-positive cells was observed in Pre- or During-NH_4_Cl-treated cells, probably because the endosomal pH was reduced enough to cause membrane fusion after the removal of NH_4_Cl, allowing cell entry of the virus. Consistent with the results that U-73122 inhibited rCl-13-ZsG multiplication, the Thrut-U-73122 treatment strongly inhibited rCl-13 infection. Intriguingly, the During-U-73122 treatment, but not the Pre- or Post-U-73122 treatment, strongly inhibited ZsG-positive cells. These observations were confirmed quantitatively by a similar time-of-addition experiment where WT rCl-13 was used instead of rCl-13-ZsG and virally infected cells were detected by flow cytometry using an antibody specific to the LCMV NP (Fig. 2c). The results indicate that LCMV infection was irreversibly inhibited by U-73122 at an early step in the LCMV life cycle.

### U-73122 impairs LCMV virion infectivity

**Fig. 3.**
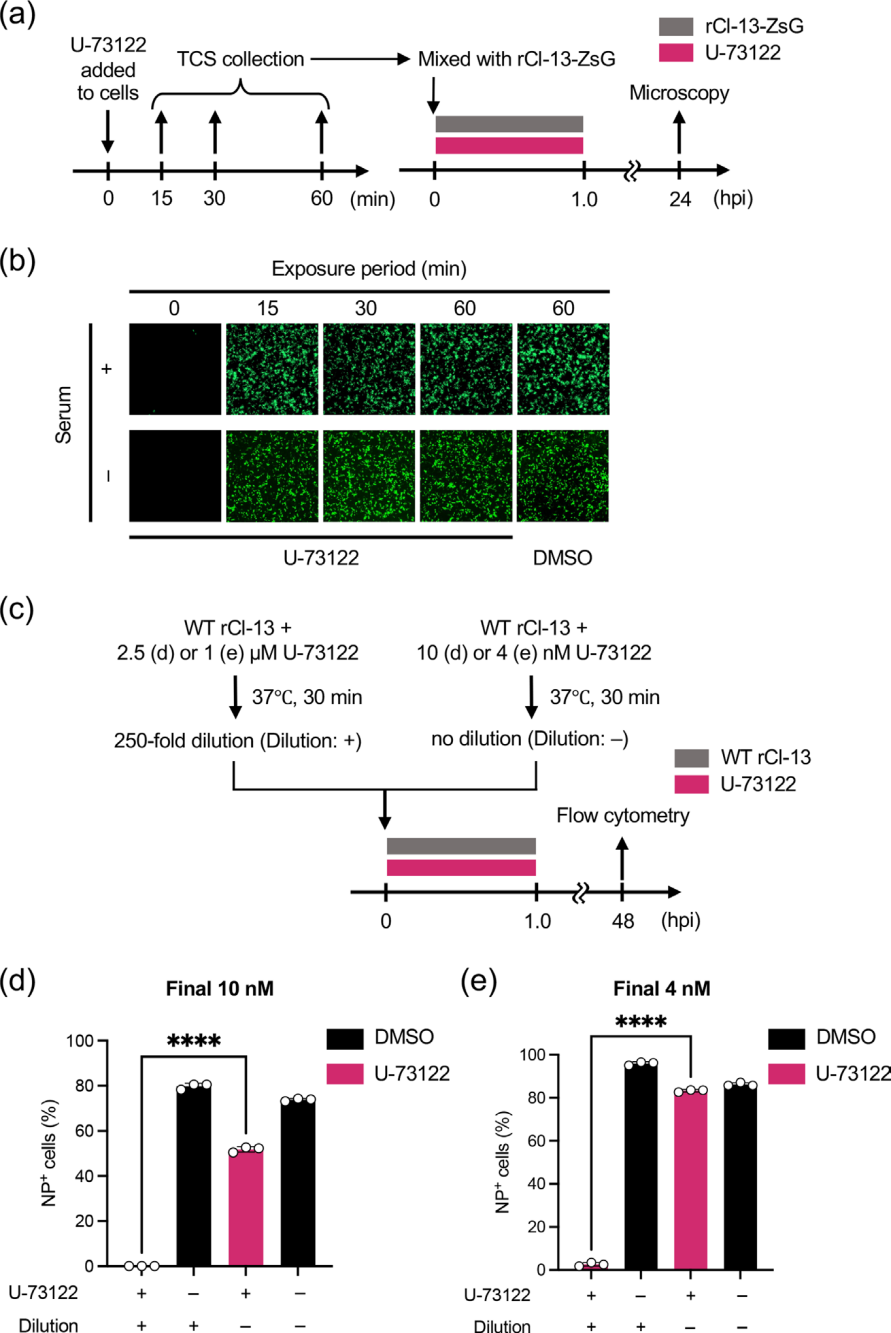
Effect of U-73122 on LCMV virion infectivity. (**a**) Flow chart of the experimental approach used to assess the durability of the anti-LCMV activity of U-73122 in TCS. For the controls, equivalent concentrations of DMSO were used instead of U-73122. (**b**) Durability of U-73122 in TCS as an LCMV inhibitor. TCSs of HEK293T cells were replaced with fresh medium containing 10 µM U-73122 or DMSO, in the presence (Serum, +) or absence (Serum, −) of serum, and the cells were incubated at 37 °C and 5% CO_2_. At the indicated time points, TCS was collected and used to prepare rCl-13-ZsG inoculum. Freshly prepared HEK293T cells seeded in a 24-well plate (2.5×10^5^ cells/well) and cultured overnight were inoculated (m.o.i.=0.1) with rCl-13-ZsG inoculum containing 5 µM U-73122 or DMSO. At 24 hpi, ZsGreen (ZsG) expression was observed with a fluorescence microscope. (**c**) Flow chart of the experimental approach used to assess the effect of U-73122 on LCMV virion infectivity. For the controls, equivalent concentrations of DMSO were used instead of U-73122. (**d, e**) Effect of U-73122 on virion infectivity. WT rCl-13 samples were incubated in medium containing 10 nM or 2.5 µM (d) or 4 nM or 1 µM (e) U-73122 (U-73122: +) or DMSO (U-73122: −). After 30-min treatment at 37 °C, viral samples containing 2.5 µM (d) and 1 µM (e) U-73122 and DMSO were diluted 250-fold. The diluted (dilution: +) or undiluted (dilution: −) virus samples were used to infect HEK293T cells (m.o.i.=0.01). The final concentrations of U-73122 were 10 nM (d) and 4 nM (e). After 60 min of adsorption, cells were washed twice and cultured in a fresh medium without compound. At 48 hpi, LCMV-NP-positive (NP^+^) cells were detected by flow cytometry. Data are the mean±sd of three independent experiments. ****P<0.0001.

In the time-of-addition experiments, the During-U-73122 treatment strongly inhibited LCMV multiplication even after the removal of U-73122. The Post-U-73122 treatment did not inhibit LCMV infection, whereas the Post-NH_4_Cl treatment inhibited the second round of infection. These results indicate that U-73122 irreversibly inhibited LCMV infection, whereas incubation of U-73122 in TCS diminished anti-LCMV activity. To determine the durability of U-73122 in TCS as an LCMV inhibitor, U-73122-containing medium (Serum, +) was added to HEK293T cells and incubated for 15, 30 and 60 min (Fig. 3a). The collected TCSs containing U-73122 were mixed with rCl-13-ZsG and used to inoculate (m.o.i.=0.1) fresh HEK293T cells. Fresh U-73122 was used to prepare the virus inoculum as a positive control (exposure period of 0 min). After 1 h of virus adsorption, virus inoculum was removed, the cells were washed with PBS and a fresh medium was added. ZsG expression was observed at 24 hpi. U-73122 did not inhibit rCl-13-ZsG infection when incubated in TCS for as short as 15 min (Fig. 3b). Similar results were obtained using serum-free medium during incubation of U-73122 with cells (Serum, −), suggesting that cellular components, rather than serum components, abolished the anti-LCMV activity of U-73122 (Fig. 3b).

The inhibitory effect of U-73122 on LCMV infection was observed consistently when U-73122 was added to the virus prior to exposure to the cells. This finding indicates that U-73122 exhibited anti-LCMV activity by binding to the virion rather than through inhibition of the PLC-*γ* activity of the cell. To examine this possibility, WT rCl-13 inoculum was prepared in the presence of 10 nM or 2.5 µM U-73122, or vehicle (DMSO) (Fig. 3c). Two vehicle controls that contained DMSO equivalent concentrations of 10 nM and 2.5 µM U-73122 were prepared. After 30-min incubation at 37 °C, the virus inoculums containing 2.5 µM U-73122 and equivalent DMSO concentration were diluted 250-fold with fresh medium to reduce the concentration of U-73122 to 10 nM. Virus inoculums that contained 10 nM U-73122 and equivalent DMSO concentration were not diluted. The diluted and undiluted virus inoculums were used to inoculate (m.o.i.=0.01) HEK293T cells for 1 h. Then, the cells were washed with PBS and a fresh medium was added. At 48 hpi, virally infected cell numbers were determined by flow cytometry using the antibody specific to LCMV NP. Incubation with 2.5 µM of U-73122 almost completely (>99 %) diminished virion infectivity even after the inoculum was diluted to 10 nM, whereas 10 nM U-73122 treatment showed only a modest (~30%) decrease in the number of NP-positive cells (Fig. 3d). To reduce U-73122 to a concentration low enough not to exhibit anti-LCMV activity, we performed a similar experiment using U-73122 at 2.5-fold lower concentration. Treatment of cell-free WT rCl-13 with 1 µM U-73122 followed by dilution to 4 nM strongly reduced virion infectivity, whereas 4 nM U-73122 treatment alone had negligible impact on LCMV infectivity (Fig. 3e). These results indicate that U-73122 bound to the virion with a low dissociation rate, which diminished virion infectivity.

### Maleimide moiety in U-73122 is essential for anti-LCMV activity

**Fig. 4.**
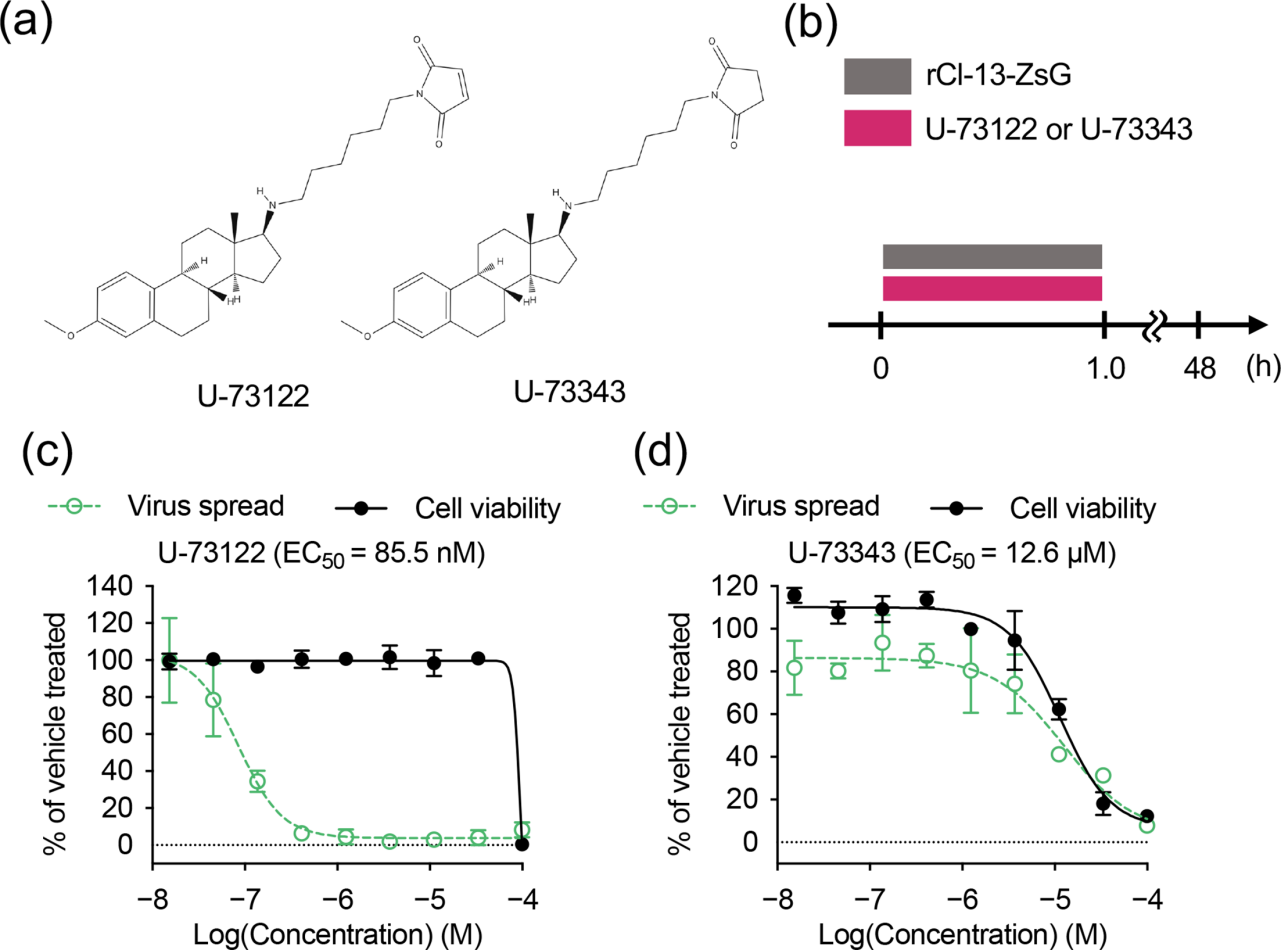
Effect of U-73343 on rCl-13 multiplication. (**a**) Structural formula of U-73122 and U-73343. (**b**) Schematic diagram of the duration of compound treatment and virus inoculation in the experiments of (c) and (d). (**c, d**) Determination of the EC_50_ and CC_50_ values. EC_50_ and CC_50_ values were determined as in Fig. 1(f), but treating and inoculating cells with U-73122 (**c**) or U-73343 (**d**) and rCl-13-ZsG for the duration indicated in (b). Data are the mean±sd of six replicates.

The irreversible inhibition of LCMV infectivity by U-73122 indicated that U-73122 may bind covalently to a molecule on the virion surface and exert anti-LCMV activity. U-73122 has a maleimide moiety that intrinsically reacts with N-based nucleophiles and thiols to form Michael addition products. To characterize the role of maleimide in U-73122 in inhibiting LCMV infectivity, we used U-73343, a close structural analogue of U-73122 that has a succinimide instead of a maleimide, which only weakly inhibited PLC [34] (Fig. 4a). We determined the specific antiviral effects of U-73122 and U-73343 under conditions similar to those that were used for the During treatment in the time-of-addition experiments. The TCS of HEK293T cells was removed, and virus inoculum containing rCl-13-ZsG (m.o.i.=0.01) and a threefold serial dilution of U-73122 or U-73343 or vehicle (DMSO) was added (Fig. 4b). Virus inoculum was removed 1 h after virus adsorption, the cells were washed with PBS and fresh medium was added. U-73122 had CC_50_ and EC_50_ values of 89.1 µM and 85.5 nM, respectively (Fig. 4c). This EC_50_ value (85.5 nM) is compatible with the EC_50_ value (79.1 nM) obtained under conditions similar to those that were used for the Thrut treatment (Fig. 1f). Unlike U-73122, U-73343 did not exhibit anti-LCMV activity and had CC_50_ and EC_50_ values of 14 µM and 12.6 µM, respectively (Fig. 4d). These results indicate that U-73122 impaired LCMV infectivity by a substitution reaction of the maleimide moiety with a nucleophilic atom (nitrogen or sulphur) in the virion.

### U-73122 inhibits the infectivity of rCl-13 expressing the glycoprotein of the parental Arm strain or LASV

**Fig. 5.**
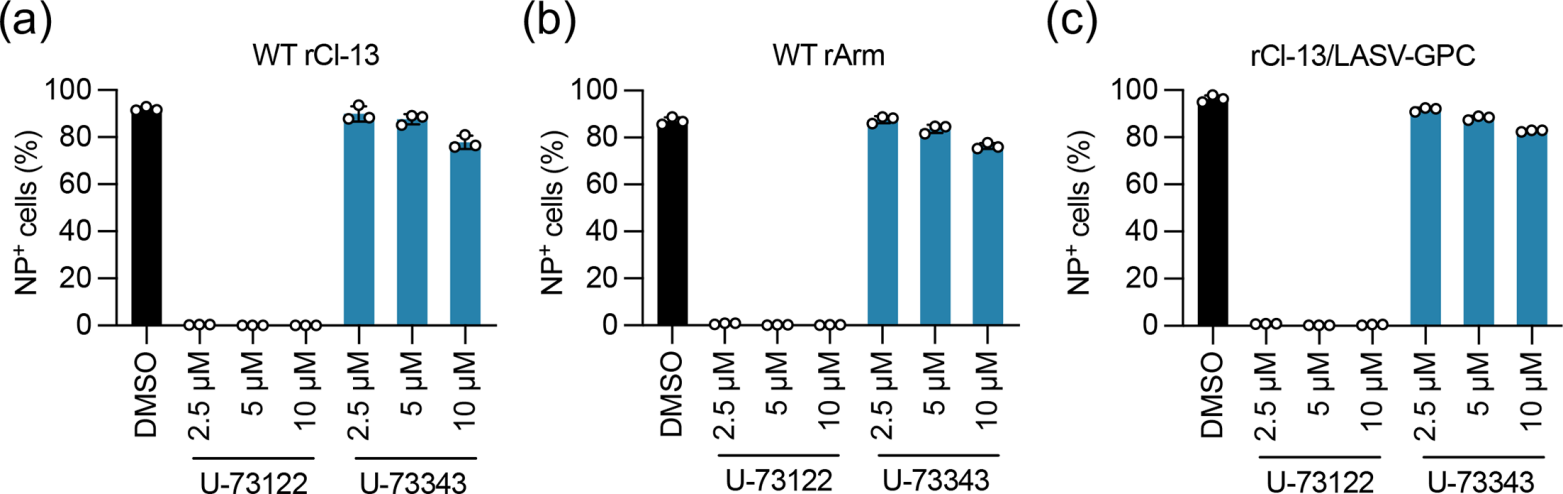
Effect of U-73122 on infection with WT rArm and rCl-13 expressing the LASV glycoprotein. (a–c) Effects of U-73122 and U-73343 on infection with LCMVs with different affinities to *α*DG. HEK293T cells seeded in a 24-well plate (2.5×10^5^ cells/well) and cultured overnight were inoculated (m.o.i.=0.1) for 1 h with WT Cl-13 (**a**), WT rArm (**b**) or rCl-13/LASV-GPC (**c**) premixed with the indicated concentrations of U-73122 or U-73343 or DMSO. The cells were washed twice with PBS and cultured in a fresh medium without compounds. At 24 hpi, LCMV-NP-positive (NP^+^) cells were detected by flow cytometry. Data are the mean±sd of three independent experiments.

A possible explanation for the impairment of LCMV infectivity by U-73122 is that U-73122 binds to the viral glycoprotein and blocks the interaction with the cell entry receptor. The Cl-13 glycoprotein had a high binding affinity to the cell surface receptor, *α*DG, whereas the glycoprotein of the parental Arm strain of LCMV had a low affinity to *α*DG [44]. To determine whether the differential affinity to *α*DG impacted the ability of U-73122 to inhibit virion infectivity, HEK293T cells were inoculated (m.o.i.=0.1) with rCl-13 or rArm containing U-73122 (2.5, 5 or 10 µM), U-73343 (2.5, 5 or 10 µM) or vehicle (DMSO) for 1 h. Then, the cells were washed with PBS and a fresh medium was added. Similar to rCl-13, U-73122 strongly inhibited rArm infection (Fig. 5a, b). Next, we investigated whether the inhibitory effect of U-73122 on LCMV infection extended to the infection mediated by the LASV glycoprotein that used a different cell entry factor, LAMP1. For this, we examined the inhibitory effect of U-73122 on rLCMV in which the GPC gene was replaced with the GPC gene of LASV (rCl-13/LASV-GPC). Similar to rCl-13 and rArm, U-73122 strongly reduced rCl-13/LASV-GPC-infected cell numbers (Fig. 5c), suggesting that the inhibitory effect of U-73122 on virion infectivity among OW mammarenaviruses was not restricted to the type of viral glycoprotein.

### LCMV-dependent virion envelope composition other than the viral glycoprotein is critical for the ability of U-73122 to diminish virion infectivity

**Fig. 6.**
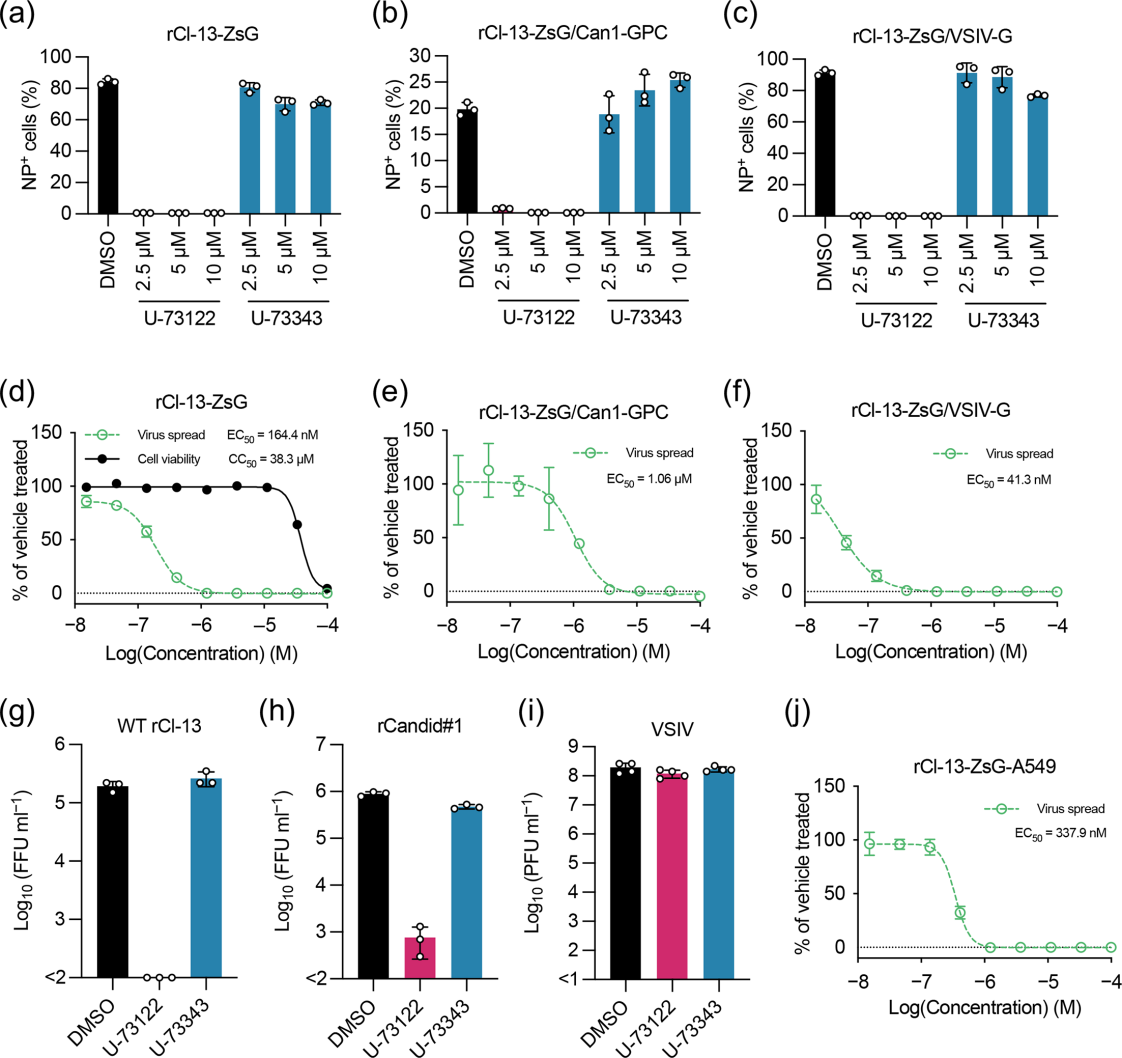
Effect of U-73122 on infection with rCl-13 expressing glycoproteins from JUNV and VSIV. (a–c) Effects of U-73122 and U-73343 on infection with rCl-13s expressing glycoproteins of distantly related viruses. Virally infected cell populations were determined as in Fig. 5(a) but inoculating cells with rCl-13-ZsG, rCl-13-ZsG/Can1-GPC or rCl-13-ZsG/VSIV-G (m.o.i.=0.01) and fixing the cells at 48 (rCl-13-ZsG and rCl-13-ZsG/Can1-GPC) or 24 (rCl-13-ZsG/VSIV-G) hpi. Data are the mean±sd of three independent experiments. (d–f) Determination of the EC_50_ and CC_50_ values. EC_50_ and CC_50_ values were determined as in Fig. 4(c) but using HEK293T cells seeded (4.5×10^4^ cells/well) a day prior to inoculation with rCl-13-ZsG (m.o.i.=0.1), rCl-13-ZsG/Can1-GPC (m.o.i.=0.01) or rCl-13-ZsG/VSIV-G (m.o.i.=0.01). The cells were fixed at 48 (rCl-13-ZsG and rCl-13-ZsG/Can1-GPC) or 24 (rCl-13-ZsG/VSIV-G) hpi. (g–i) Viral titres in TCSs of cells treated with U-73122 or U-73343. HEK293T cells seeded (2.5×10^5^ cells/well) in a 24-well plate and cultured overnight were inoculated for 1 h with WT rCl-13 (m.o.i.=0.1), rCandid#1 (m.o.i.=0.1) or VSIV (m.o.i.=1) premixed with U-73122 (2.5 µM), U-73343 (2.5 µM) or DMSO. Cells were washed twice with PBS and cultured in fresh medium without compounds. At 24 (WT rCl-13), 48 (rCandid#1) or 9 (VSIV) hpi, viral titres in TCSs were determined by immunofocus-forming assay or plaque assay. FFU, focus-forming unit; PFU, plaque-forming unit. Data are the mean±sd of three (WT rCl-13 and rCandid#1) or four (VSIV) independent experiments. (**j**) Determination of the EC_50_ value for rCl-13-ZsG-A549. The EC_50_ value was determined as in Fig. 6(d), using rCl-13-ZsG that was twice passaged in A549 cells.

We next asked whether U-73122 inhibited LCMV infection mediated by glycoproteins of NW mammarenavirus, JUNV, and a genetically distantly related RNA virus, VSIV. For this, we used rCl-13-ZsGs expressing the glycoprotein of Candid#1 (rCl-13-ZsG/Can1-GPC) or the VSIV glycoprotein (rCl-13-ZsG/VSIV-G) instead of Cl-13-GPC. HEK293T cells were inoculated (m.o.i.=0.01) with rCl-13-ZsG, rCl-13-ZsG/Can1-GPC or rCl-13-ZsG/VSIV-G containing U-73122 (2.5, 5 or 10 µM), U-73343 (2.5, 5 or 10 µM) or vehicle (DMSO) for 1 h. Then, the cells were washed with PBS and a fresh medium was added. At 48 hpi with rCl-13-ZsG and rCl-13-ZsG/Can1-GPC or at 24 hpi with rCl-13-ZsG-VSIV-G, the cells were fixed and virally infected cells were determined by flow cytometry. Besides rCl-13-ZsG, U-73122, but not U-73343, strongly inhibited infection of rCl-13-ZsG/Can1-GPC and rCl-13-ZsG/VSIV-G (Fig. 6a–c). Inhibitory effects of U-73122 on rCl-13 with different viral glycoproteins were examined by determining EC_50_ values under conditions that were similar to those used for the former experiments (Fig. 6a–c). Under these conditions, the CC_50_ of U-73122 was 38.3 µM (Fig. 6d). U-73122 strongly inhibited rCl-13-ZsG/Can1-GPC multiplication with an EC_50_ of 1.06 µM, albeit higher than the EC_50_ of U-73122 on rCl-13-ZsG (164.4 nM) (Fig. 6d, e). The EC_50_ of U-73122 on rCl-13-ZsG/VSIV-G (41.3 nM) was even lower than that on rCl-13-ZsG (Fig. 6f). These results suggest that U-73122 diminished virion infectivity by binding to a molecule incorporated into the LCMV virion, but not to the viral glycoprotein. To investigate whether virion composition affected the sensitivity to U-73122, we examined the inhibitory effect of U-73122 on NW JUNV (Candid#1 strain) and VSIV. rCl-13, rCandid#1 and VSIV inoculums containing 2.5 µM U-73122 or U-73343 or vehicle (DMSO) were added (m.o.i.=0.1 for rCl-13 and rCandid#1; m.o.i.=1 for VSIV) to HEK293T cells. After 1-h adsorption of virus inoculum, the cells were washed with PBS and fresh medium was added. At 24 (rCl-13), 48 (rCandid#1) and 9 (VSIV) hpi, viral titres of TCS were examined. Similar to rCl-13 (Fig. 6g), rCandid#1 production was strongly inhibited by U-73122 but not by U-73343 (Fig. 6h), whereas VSIV production was not inhibited by U-73122 or U-73343 (Fig. 6i). The virion envelope composition can vary depending on the cell type used to produce the virus. To assess whether anti-LCMV activity of U-73122 was significantly influenced by the cell type used to produce the virus, rCl-13-ZsG was twice passaged in A549 cells at a low m.o.i. (0.01), and the resulting virus rCl-13-ZsG-A549 was used to determine the EC_50_, as was done for rCl-13-ZsG, which was expanded in BHK-21 cells (Fig. 6d). U-73122 retained a comparable level of inhibition against rCl-13-ZsG-A549 infectivity (EC_50_=337 nM), implying that U-73122 exerts its anti-LCMV activity through an association with a molecule on the LCMV virion commonly incorporated from host cells (Fig. 6j).

## Discussion

In a previous study, we demonstrated that the ErbB signalling pathway contributed to the multiple steps of the life cycle of mammarenaviruses, but U-73122, an inhibitor of PLC-*γ* that acts downstream of ErbB signalling, did not inhibit LASV or LCMV minigenome activity. The lack of inhibitory effect of U-73122 on vRNP activity led us to explore the possibility that PLC-*γ* contributed to a step in the mammarenavirus life cycle other than the transcription and replication of the viral genome. U-73122 strongly and irreversibly inhibited LCMV infection; however, unexpectedly, the mechanism of action of U-73122 involved the disruption of virion infectivity rather than the inhibition of PLC-*γ* in cells. U-73122, 1-[6-((17*β*-3-methoxyestra-1,3,5(10)-trien-17-yl)amino)hexyl]-1H-pyrrole-2,5-dione, contains a six-carbon chain between the maleimide and estrone scaffold. A simple explanation for the action of U-73122 on anti-LCMV activity is that the hydrophobic carbon chain possibly sticks into viral envelope lipids, leading to the decay of virions. However, this action seems unlikely because U-73122 (2.5 µM) did not inhibit infection of VSIV, which also has a lipid bilayer membrane.

U-73122 (2.5 µM) strongly inhibited rCl-13-ZsG/VSIV-G but not VSIV, indicating that U-73122 impaired LCMV virion infectivity by binding to a molecule incorporated into the LCMV virion but not the VSIV virion. Enveloped viruses uptake viral proteins and host cellular proteins into virions. Some of these proteins may be coincidentally incorporated, but others have been reported to play roles in efficient viral production and subsequent infections. The host proteins required for viral assembly and budding may accumulate at the site of viral egress and are highly likely to be incorporated into the virion. To determine the host–cell interaction required for virion production, the proteome of purified severe acute respiratory syndrome coronavirus 2 (SARS-CoV-2) has been analysed [45]. Among the 92 virion-associated host proteins, G3BP1 and G3BP2, key molecules for the formation of stress granules [46,49], contributed to the formation of the local environment, favouring the assembly of viral components for efficient SARS-CoV-2 particle formation. Intriguingly, some of the host proteins that were incorporated into the herpes simplex virus 1 (HSV-1) virion were reported to play a proviral role in the next round of infection [50]. Stegen *et al.* investigated the impact of small interfering RNA-mediated gene knockdown against 49 previously identified host proteins associated with the HSV-1 virion on HSV-1 multiplication. This screening identified 15 of the 49 host proteins for which the depletion significantly inhibited the production of infectious HSV-1 progeny. Moreover, 13 of the 15 host proteins were required for efficient viral production in new infections because viral yields were significantly reduced in normal cells inoculated with HSV-1 particles released from cells in which each of the 13 host proteins was knocked down. The results of the present study indicate that U-73122 binds to an LCMV virion-associated molecule that may have a pivotal role in the next round of infection. Characterization of the proteome of the LCMV virion and identification of the target protein of U-73122 will advance the understanding of the roles of host cell proteins incorporated into the LCMV virion in subsequent infections.

VSIV has a high density of surface VSIV-G protein [51], whereas the rCl-13-ZsG/VSIV-G virion may have a lower density of VSIV-G compared with that of VSIV potentially because of the absence of specific interactions with other viral proteins necessary for efficient virion assembly [52]. This, together with the different susceptibilities of VSIV and rCl-13/VSIV-G to U-73122, indicates that surface glycoprotein density may influence the accessibility of U-73122 to bind to a molecule on the virion, thus affecting its antiviral activity. A detailed assessment of the antiviral spectrum of U-73122 using a series of enveloped viruses with varying glycoprotein densities, including human immunodeficiency virus 1 [53] and SARS-CoV-2 [54], as examples of low-density surface spike protein viruses, and influenza A virus [55], as an example of a high-density surface spike protein virus, may provide insights into the relevance of surface glycoprotein density to antiviral activity of U-73122. Such studies may also discover whether the target molecule of U-73122 is specifically incorporated into the virions of mammarenaviruses or is more broadly incorporated across enveloped viruses, informing the potential spectrum of antivirals that could be developed by targeting the molecule incorporated into virions.

It is plausible that U-73122 binds covalently to lipids that have amines such as phosphatidylserine (PS) and phosphatidylethanolamine. Enhancement of viral cell entry by binding of virion-associated PS to PS receptors on the cell has been demonstrated in several enveloped viruses [56]. Among the variety of PS receptors with differential biological properties [57], the relevance of the T cell, immunoglobulin and mucin (TIM) family and the Tyro3, Axl and Mer family of PS receptors in cell entry of enveloped viruses has been well studied. The contribution of PS receptors in medicating cell entry of OW mammarenaviruses, LASV and LCMV, has been observed depending on the experimental conditions. Human fibrosarcoma HT-1080 cells lack the glycosyltransferase-like acetylglucosaminyltransferase (LARGE) required for O-glycosylation of *α*DG, which is essential for binding to the LASV glycoprotein [58,59]. Infection of HT-1080 cells with a lentivirus pseudotyped with LASV glycoprotein or chimeric rLCMV expressing LASV-GPC was inhibited by Axl antibody [60,61]. Double knockdown of DG and Axl in HT-1080 cells significantly reduced cell numbers infected with the chimeric virus [60]. Overexpression of Axl or Tyro3 resulted in enhanced susceptibility of Jurkat cells deficient in LARGE-glycosylated *α*DG to lentiviruses pseudotyped with LASV or LCMV glycoprotein [61,62]. Similarly, the entry of VSIV-based LASV glycoprotein pseudovirion to Vero cells that do not express glycosylated DG compatible with LASV glycoprotein binding was significantly inhibited by gene knockout of TIM-1. These findings indicate that PS receptors mediate *α*DG-independent cell entry of OW mammarenaviruses in cultured cells. However, the significance of PS receptors in *in vivo* infection is not evident because LCMV normally propagates in Axl knockout mice [63]. In the current study, we used HEK293T cells that express fully glycosylated *α*DG, and cell entry of LCMV to HEK293T cells may not be mediated by PS receptors. U-73122 also inhibited infection with rLCMV expressing VSIV-G, whereas VSIV-G-mediated cell entry was not affected by overexpression or suppression of PS receptors [60,61]. Therefore, U-73122 binding to virion-associated PS may not account for the anti-LCMV activity of U-73122.

U-73343, 1-[6-((17*β*-3-methoxyestra-1,3,5(10)-trien-17-yl)amino)hexyl]-2,5-pyrrolidinedione, is a chemical analogue of U-73122 in which the maleimide group is replaced by a succinimide group. Unlike U-73122, the inhibition of LCMV infectivity by U-73343 was not observed, implying that the reactivity of maleimide as a Michael acceptor may be involved in the antiviral activity of U-73122. U-73122 was reported to bind covalently to several cystine residues of PLC-*β*3, a PLC variant, but the cystine residue responsible for the inhibition of PLC was not determined [64]. In addition to PLC-*γ*, U-73122 inhibited 5-lipoxygenase (5-LO) [65], an enzyme that plays an important role in the biosynthesis of leukotrienes, eicosanoid lipid mediators [66]. A matrix-assisted laser desorption/ionization mass spectrometry analysis showed that U-73122 covalently bound to cysteine residues 99, 159, 248, 264, 416 and 449 of 5-LO [67]; among them, cysteine 416 was essential for potent inhibition of 5-LO by U-73122. It is plausible that U-73122 binds covalently to surface-exposed cysteine residues of the LCMV virion-associated protein to irreversibly impair virion infectivity. Interestingly, the depletion of cellular thiols by the addition of the thiol-reactive compound *N*-ethylmaleimide significantly enhanced the activity of U-73122 in 5-LO inhibition [65]. Besides PLC, U-73122 is also a potent inhibitor of the histamine H_1_ receptor [68], telomerase [69], the sarcoplasmic/endoplasmic reticulum calcium ATPase pump in smooth muscle [70] and the adenosine A_1_ receptor [71]. Moreover, U-73122 formed conjugates with glutamine, glutathione and BSA [72]. Together, these findings indicate that the target specificity of U-73122 is less stringent, and therefore, U-73122 with an electrophilic maleimide group was quickly trapped by nucleophilic molecules, resulting in the short duration of anti-viral activity of U-73122 in TCS.

Regardless of the high reactivity and obscure target specificity of U-73122, VSIV infection was not inhibited by U-73122 at 2.5 µM. Because of the high VSIV stock titre, the virus solution was diluted approximately 400-fold when it was mixed with U-73122. Therefore, the effects of components derived from the VSIV stock solution other than infectious virions on U-73122 activity would have been negligible. However, a limitation of our study is that although we diluted the virus solution, we could not remove virion-non-associated components from the virus solution, which may have led to an underestimation of the antiviral activity of U-73122. Further investigation of the properties of molecules preferentially targeted by U-73122 will allow a more precise assessment of the levels of confounding factors and a more accurate evaluation of the bona fide antiviral activity of U-73122.

Interestingly, *N*-ethylmaleimide itself had no potent inhibitory activity against 5-LO [65], and U-73343, in contrast to human 5-LO, inhibited the activity of 5-LO in mice and rats [67], suggesting that both the maleimide moiety and the rest of the U-73122 structure are important determinants of the biological activity of U-73122. Despite the potent anti-LCMV activity of U-73122, the short duration of its antiviral activity prevented us from investigating U-73122 as a potential antiviral candidate in the context of multiple rounds of infection. A comprehensive analysis of the structure–activity relationship in the anti-LCMV activity of U-73122 will enable the chemical modification of U-73122 to improve the balance between potency and target specificity and allow U-73122 derivatives to be used for the development of U-73122-based countermeasures against human pathogenic mammarenavirus infections.

## Abbreviations

Arm: Armstrong
ATCC: American Type Culture Collection
CC_50_: 50% cytotoxic concentration
Cl-13: clone 13
DMEM: Dulbecco’s modified Eagle medium
EC_50_: 50% effective concentration
GPC: glycoprotein precursor
hpi: h post-inoculation
HSV-1: herpes simplex virus 1
JUNV: Junín virus
LASV: Lassa virus
LCMV: lymphocytic choriomeningitis virus
5-LO: 5-lipoxygenase
NP: nucleoprotein
NW: New World
OW: Old World
PFA: paraformaldehyde
PLC: phospholipase C
PS: phosphatidylserine
r: recombinant
SARS-CoV-2: severe acute respiratory syndrome coronavirus 2
SI: selectivity index
TCS: tissue culture supernatant
TIM: T cell, immunoglobulin and mucin
vRNP: viral ribonucleoprotein
VSIV: vesicular stomatitis Indiana virus
VSIV-G: vesicular stomatitis Indiana virus glycoprotein
*α*DG: *α*-dystroglycan
